# Levels and Determinants of Antenatal Breastfeeding Attitudes among Pregnant Women: A Cross-Sectional Study

**DOI:** 10.3390/children10020275

**Published:** 2023-01-31

**Authors:** Li Liu, Gui Xiao, Tingting Zhang, Mengjia Zhou, Xingxing Li, Yu Zhang, Theresah Owusua, Yang Chen, Chunxiang Qin

**Affiliations:** 1 Department of Health Management, Department of Nursing, The Third Xiangya Hospital, Central South University, Changsha 410013, China; 2 Xiangya School of Nursing, Central South University, Changsha 410013, China; 3 School of Medicine, Jishou University, Jishou 416000, China; 4 School of Nursing and Midwifery, Catholic University College of Ghana, Sunyani 363, Ghana; 5 Department of Nursing, The Third Xiangya Hospital, Central South University, Changsha 410013, China

**Keywords:** breastfeeding attitudes, determinants, antenatal depressive symptoms

## Abstract

Breastfeeding attitudes are strong predictors of breastfeeding behavior. Gaining a deeper understanding on the levels and determinants of antenatal breastfeeding attitudes is crucial. This cross-sectional study involved 124 pregnant women at a tertiary hospital in Hunan, China. A self-administered questionnaire, the Iowa Infant Feeding Attitude Scale, the Edinburgh Postnatal Depression Scale, the Pregnancy Stress Rating Scale, the Childbirth Attitude Questionnaire, the Perceived Social Support Scale, and the Breastfeeding Knowledge Questionnaire were assessed during their first-trimester, second-trimester, and third-trimester hospital visit. Multiple linear regression was conducted to identify the determinants of breastfeeding attitudes. The participants reported neutral (56.39 ± 5.69) levels of breastfeeding attitudes. The determinants of antenatal breastfeeding attitudes were other family members’ support for exclusive breastfeeding: moderate (β = 0.278, *p* < 0.05), depressive symptoms (β = −0.191, *p* < 0.05), and breastfeeding knowledge (β = 0.434, *p* < 0.001). The variables explained 33.9% (adjusted R^2^) of the total variation in breastfeeding attitudes scores (F = 4.507, *p* < 0.001). Namely, other family members’ support for EBF was a negative influence on positive breastfeeding attitudes. The women whose other family members were moderate of EBF had more positive attitudes toward breastfeeding compared to those whose other family members were very supportive of EBF. The depressive symptoms were negatively associated with positive breastfeeding attitudes, and lower levels of depressive symptoms were associated with higher levels of positive breastfeeding attitudes among pregnant women. Additionally, breastfeeding knowledge was positively associated with positive breastfeeding attitudes. The more knowledgeable about breastfeeding, the more positive the attitude towards breastfeeding. Health professionals should identify these modifiable factors that may contribute to poorer breastfeeding attitudes, which is useful in targeting promotions of breastfeeding.

## 1. Introduction

The World Health Organization (WHO) recommends that mothers exclusively breastfeed their children for 6 months, with a goal of breastfeeding for up to 2 years of age and beyond, but few women achieved it [1,2]. The global exclusive breastfeeding (EBF) rate for infants at 6 months is 43% [2]. A study has found that only 37% of infants under six months in low- and middle-income countries were exclusively breastfed [3], and the rate of EBF for up to 6 months in China was only 29.2% [4]. Breastfeeding attitudes refer to women’s tendency to express their feelings, thoughts, and behaviors related to mental mindfulness as they develop their subsequent EBF behaviors [5]. As a modifiable element, breastfeeding attitudes are one of the strategic intervention directions to improve breastfeeding [6]. Previous researches have demonstrated that breastfeeding attitudes are important predictors of choice of infant feeding practices and EBF duration [7,8]. It has been reported that 82–97% of women make their decision about EBF during pregnancy [9,10]. Of those who have favorable attitudes towards breastfeeding at this point (75–97%), the majority initiate breastfeeding after delivery [11,12]. Therefore, investigating the levels of antenatal breastfeeding attitudes and finding factors that are associated with negative maternal breastfeeding attitudes is important for developing intervention strategies to promote postpartum breastfeeding behaviors.

Our literature review found that breastfeeding attitudes might be affected by maternal age, residence, race/ethnicity, educational status, occupation, marital status, household income, pregnancy intention, breastfeeding intention, breastfeeding knowledge, and social support, etc. [8,13,14,15,16,17,18,19,20,21]. When evaluated in this respect, it is affected not only by the socio-demographic and EBF-related factors mentioned above, but also by one’s perinatal mental health. For example, recent studies reported that depressed and stressed mothers might be poorer in breastfeeding attitudes. Some research found that women with depressive symptoms were more likely to have a negative attitude towards breastfeeding [13,15,18,22,23,24]. Additionally, the research of Duran et al. showed that the mothers’ positive breastfeeding attitudes decreased as their perceived stress levels increased in the postpartum period [16]. Nevertheless, most of the published literature on the relationship between breastfeeding attitudes and mental health focuses on the postpartum period, with few studies mentioning the relation between psychological factors during pregnancy and antenatal breastfeeding attitudes [22].

Pregnancy is most often a period of mental well-being for women. After the drop in hormones during childbirth, there are disturbances in the woman’s mental health. In addition, family roles change and more responsibilities are assigned to the couple. Therefore, it is a period of increased vulnerability to emotional changes, such as depressive symptoms (roughly 20.7% prevalence), pregnancy stress (roughly 94.4% prevalence), and fear of childbirth (6.3–14.8% prevalence) [25,26,27]. These psychological problems have been well documented to have serious adverse impacts on the health of mothers and babies and they may be potential risk factors for breastfeeding attitudes. Identifying these psychological factors that may lead to poorer breastfeeding attitudes, especially when they can be modified, is a high research priority.

In conclusion, further research on breastfeeding attitudes that comprehensively consider antenatal psychological, EBF-related, and socio-demographic factors is needed. A deeper understanding of the levels and specific determinants of antenatal breastfeeding attitudes is essential for policymakers to design district-appropriate targeted interventions to encourage mothers to prepare for breastfeeding. Thus, this study aims to investigate the levels and determinants of antenatal breastfeeding attitudes among pregnant women.

## 2. Materials and Methods

### 2.1. Design and Participants

This was a cross-sectional study which was performed from October 2021 to July 2022 in a hospital in Hunan Province, China. The inclusion and exclusion criteria for the participants were as follows. The inclusion criteria included mothers with their first spontaneous pregnancy, older than 18 years, with a singleton gestation of 8–13 weeks’ gestation, and no contraindications to restrict breastfeeding. Additionally, the exclusion criteria included the participants with suicidal tendencies or severe mental illness or who refused to participate in this study.

Sample size calculations (using G*Power version 3.1 [28]) determined that 93 participants were required to provide power (1 − β = 0.80) to examine medium effect size (*f*^2^ = 0.15) correlations using multiple linear regression analyses with five hypothesized predictors (EBF intention, breastfeeding knowledge, depressive symptoms, pregnancy stress, and fear of childbirth). Additionally, the α was 0.05. Together with a possible 20% no-response rate, the minimum sample size required was 110.

### 2.2. Measures

Our primary outcome was breastfeeding attitude. The potential influence factors were the participant characteristics, antenatal depressive symptoms, pregnancy stress, fear of childbirth, breastfeeding knowledge, and social support. The specific measurement tools are shown below.

#### 2.2.1. Participant Characteristics

The pregnant women were asked to complete a self-administered questionnaire. It included socio-demographic and EBF-related characteristics. The following descriptive characteristics of the participants were obtained: age, residence, race/ethnicity, educational status, occupation, marital status, household income, pregnancy intention and EBF intention, and spousal and other family members’ support for EBF. The definition of breastfeeding-related in this study strictly follows the WHOs guidelines [29].

#### 2.2.2. Breastfeeding Attitude

The Iowa Infant Feeding Attitude Scale (IIFAS) was used to assess breastfeeding attitudes [30]. The IIFAS comprises 17 items, including a variety of dimensions of infant feeding attitudes. It uses a five-point Likert scale, with a score of 1 for strongly disagree and 5 for strongly agree. The score range was 17 to 85, with higher scores favoring breastfeeding. The infant feeding attitude score could be divided into three levels according to the score: (1) positive to breastfeeding (70–85), (2) neutral (49–69), and (3) positive to formula feeding (17–48) [30]. This instrument has been introduced in several countries and regions and has been applied in different populations. The Cronbach ‘s α of the Chinese version was 0.62 [31]. The Cronbach’s α was 0.63.

#### 2.2.3. Antenatal Depressive Symptoms

The Edinburgh Postnatal Depression Scale (EPDS) was used to measure antenatal depressive symptoms [32]. The scale was originally developed specifically for postpartum depression but is commonly used to measure levels of depressive symptoms during all periods of the perinatal period because of its simplicity, understandability, and ease of maternal acceptance. It is a 10-item scale, which mothers were instructed to rank on a four-point Likert scale. The total score ranges from 0 to 30. The higher the score, the more severe the depressive symptoms. The Chinese version of EPDS with satisfactory content validity and reliability was adopted (Cronbach’s α was 0.78) [33,34]. In our study, the Cronbach’s α was 0.80.

#### 2.2.4. Pregnancy Stress

The Pregnancy Stress Rating Scale (PSRS) was used to measure pregnancy stress. It is a commonly measured antenatal stress scale and mainly used to evaluate the source of stress in pregnant women and the degree of its impact. The PSRS comprises four stressors. Each item is divided into four levels: “not at all”, “a little”, “often”, and “always”. The total score range from 0 to 90, with a higher score indicating a higher level of pregnancy stress. A Chinese version with a good content validity and reliability was adopted (Cronbach’s α was 0.84) [35]. The Cronbach’s α in our study was 0.95.

#### 2.2.5. Fear of Childbirth

The Childbirth Attitude Questionnaire (CAQ) was used to measure fear of childbirth [36]. This scale has 16 items and includes 4 dimensions. It uses a Likert 4-point scale for each item, with a total score of 16 to 64. Higher scores indicate a more severe fear of childbirth. A Chinese version was compiled and revised by Chinese scholars [37]. Additionally, they showed a satisfactory internal consistency reliability and validity for the questionnaire (Cronbach’s α was 0.91). The Cronbach’s α was 0.95.

#### 2.2.6. Breastfeeding Knowledge

Breastfeeding knowledge was assessed using the Chinese Breastfeeding Knowledge Questionnaire (BKQ), which was compiled by Zhao, M. [38]. The BKQ consisted of 17 statements covering the benefits and skills related to breastfeeding. Each item scored 1 point for a correct answer and 0 points for an incorrect or uncertain answer. The total score is 0~17. The higher the score, the higher the level of maternal breastfeeding knowledge. The Cronbach’s α of it was 0.91 [38]. The Cronbach’s α in our study was 0.87.

#### 2.2.7. Social Support

Social support was evaluated by the Perceived Social Support Scale (PSSS) [39]. The PSSS is designed to evaluate the degree of support that individuals perceive from various sources of social support such as family, friends, and others. It uses a seven-point scale from 1 to 7 for each item, with a total of 12 items. The higher the score, the higher the level of social support perceived by the individual. Clinical applications have shown that the Chinese version of PSSS has satisfactory content validity and reliability (Cronbach’s α was 0.89) [40]. In this study, the Cronbach’s α was 0.95.

### 2.3. Data Collection

Participants were approached by members of the research team in a room of the obstetric clinic. The researchers introduced the potential participants to the main content and the purpose of our study before the survey. Then, the women were invited to participate in the investigations. Additionally, after obtaining consent from the pregnant women and signing the informed consent form, a link to the electronic questionnaire was sent to eligible pregnant women through Wechat messages. They could complete the questionnaire at their convenience.

In this study, the administration of the study questionnaires was spread over three time periods to reduce the burden on the participants [22]. In detail, the participants were invited to finish questionnaires at three time points: 8–13 weeks of gestation (Time 1, the first trimester), 22–26 weeks of gestation (Time 2, the second trimester) and 34–38 weeks of gestation (Time 3, the third trimester). At Time 1, they completed the self-administered questionnaire and the PSSS. At Time 2, they completed the PSRS and the EPDS. Additionally, at Time 3, they completed the IIFAS, the CAQ, and the BKQ. Each questionnaire took approximately ten minutes to finish. The participants could only successfully submit the questionnaire if they answered all items in the questionnaire completely. the Submitted questionnaires were reviewed by the researcher. If there were any questions, the participants were contacted immediately to confirm the information.

### 2.4. Data Analysis

SPSS version 22.0 was used to perform the data analysis. For the participants’ characteristics, continuous variables were expressed as the mean (standard deviation, SD). Additionally, categorical variables were expressed as frequencies. Statistical significance was set at two-sided *p* < 0.05. The univariate effects of the factors were analyzed by independent samples t-tests and one-way ANOVAs or Kruskal–Wallis H test. In addition, Pearson correlations and spearman correlations were used to explore the correlations between breastfeeding attitudes, depressive symptoms, pregnancy stress, fear of childbirth, breastfeeding knowledge, and social support. Finally, the variable with *p* < 0.15 from univariate analyses and correlation analysis were included in multiple linear regression analyses for testing to determine the factors influencing breastfeeding attitudes.

### 2.5. Ethical Considerations

We obtained permission from the Ethics Review Committee of Xiangya School of Nursing, Central South University (number: E202159). Detailed informed written consents were provided and explained to the participants. All participants were guaranteed the right to leave the study at any time without giving a reason or explaining why. All data collection instruments did not have any potential adverse effects on them.

## 3. Results

### 3.1. The Participants’ Characteristics

Table 1 describes the characteristics of the participants and the distribution of respondents and variables. Between October 2021 and December 2021, 178 women were approached and 159 women consented to participate in this study. After excluding 3 ineligible women, 156 pregnant women were included. As of July 2022, 124 of them who completed all three questionnaires were included in the analysis (79.5% response rate). In total, 97.6% of them were younger than 35 years [mean age (M) = 28.29, SD = 2.92], 91.1% lived in an urban district, 90.3% had Junior college degrees or above, 87.9% were employed, 95.2% were married, 78.2% had a per capita monthly household income greater than CNY 5000, and 82.3% were of a planned or natural pregnancy.

The mean value of the participants in IIFAS was 56.39 ± 5.69 (Min: 45, Max: 70). Additionally, 6.45 ± 3.99, 19.36 ± 13.77, 35.90 ± 10.30, 10.56 ± 4.40, and 69.87 ± 11.14, respectively, in EPDS, PSRS, CAQ, BKQ, and PSSS (Table 2).

### 3.2. Univariate Analysis

Table 3 reports the results of the univariate analysis. There was a significant difference in breastfeeding attitudes scores according to the EBF intention (F = 10.417, *p* < 0.05). The variables with *p* < 0.15 were residence (t = −1.721, *p* = 0.088), educational status (F = 7.693, *p* = 0.103), household income (F = 5.119, *p* = 0.077), spousal support for EBF (F = 5.327, *p* = 0.070), and other family members’ support for EBF (F = 4.427, *p* = 0.109).

### 3.3. Correlation Analysis

Table 4 shows the results of the correlation analysis. It revealed that breastfeeding attitudes were positively associated with breastfeeding knowledge (r = 0.505, *p* < 0.001). The relationships between breastfeeding attitudes and depressive symptoms (r = −0.134, *p* = 0.139), pregnancy stress (r = −0.126, *p* = 0.162), fear of childbirth (r = −0.082, *p* = 0.365), and social support (r = 0.145, *p* = 0.108) were insignificant.

### 3.4. Multiple Linear Regression Analyses

The variables with *p* < 0.15 in univariate analyses and correlation analysis (Table 3 and Table 4) were included in the multiple linear regression analysis (Table 5). The analysis revealed that the determinants of antenatal breastfeeding attitudes were other family members’ support for exclusive breastfeeding: moderate (β = 0.278, *p* < 0.05), depressive symptoms (β = −0.191, *p* < 0.05), and breastfeeding knowledge (β = 0.434, *p* < 0.001). Namely, other family members’ support for EBF was a negative influence on positive breastfeeding attitudes. The women whose other family members were moderately supportive of EBF had more positive attitudes toward breastfeeding compared to those whose other family members were very supportive of EBF. The depressive symptoms were negatively associated with positive breastfeeding attitudes, and lower levels of depressive symptoms were associated with higher levels of positive breastfeeding attitudes among pregnant women. Additionally, breastfeeding knowledge were positively associated with positive breastfeeding attitudes. The more knowledgeable about breastfeeding, the more positive the attitude towards breastfeeding. The variables explained 33.9% (adjusted R^2^) of the total variation in breastfeeding attitudes scores (F = 4.507, *p* < 0.001).

## 4. Discussion

This study showed that the antenatal breastfeeding attitudes of pregnant women in Changsha, China were at a neutral level. Additionally, depressive symptoms, breastfeeding knowledge, and other family members’ support for EBF are determinants of antenatal breastfeeding attitudes. In addition, surprisingly, other family members’ support for EBF was a negative influence on positive breastfeeding attitudes.

In this study, the mean breastfeeding attitudes score (56.39 ± 5.69) for pregnant women living in Hunan, China was generally consistent with the pregnant women living in Tianjin, China (59.8 ± 7.63), they were higher than pregnant women living in Ethiopia (54.7 ± 3.12) but lower than pregnant women in most developed countries, such as Canada (64.00 ± 10.40), the UK (66.81 ± 10.47), the USA (67.40 ± 7.30), and Greece (70.00 ± 7.60) [20,41,42,43,44,45]. Studies suggest that breastfeeding attitudes scores of 70–85 indicate a positive attitude toward breastfeed, scores of 49–69 indicate a neutral attitude, and scores of 17–48 indicate a negative attitude toward breastfeed [30]. The positive attitudes toward antenatal breastfeeding were the strongest predictors and were related to a 20–30% increase in postnatal breastfeeding initiation and maintenance rates [22,46,47]. The neutral attitude of pregnant women implied that they did not have a determined preference for exclusive breastfeeding or formula feeding. A neutral score of IIFAS also predicts a shorter duration of exclusive breastfeeding [30]. Thus, a more immediate intervention should target this group of Chinese women whose attitudes toward breastfeeding are mixed or ambivalent. Future research to develop and evaluate breastfeeding promotion programs should pay attention to breastfeeding attitudes from the antenatal period.

Our study firstly revealed depressive symptoms as an important determinant of antenatal breastfeeding attitudes for pregnant women. In the multiple linear regression analyses, antenatal depressive symptoms were significantly and negatively associated with positive breastfeeding attitudes, and lower levels of depressive symptoms were associated with higher levels of positive breastfeeding attitudes among pregnant women. Nevertheless, the results of our correlation analysis of depressive symptoms and breastfeeding attitudes were not statistically significant. This indicated that the negative effect of depressive symptoms might be masked by other factors. In addition, this inconsistency may be also related to the presence of the other potential confounders and mediating or moderating variables between the independent and dependent variables [48,49], which deserves further exploration. Meanwhile, it suggests that future research needs to pay attention to the negative effect of antenatal depressive symptoms on positive breastfeeding attitudes. Additionally, confounders need to be considered as comprehensively as possible when conducting such studies. Previous studies also reported the negative relationship between positive breastfeeding attitudes and depressive symptoms, but they were conducted in the postpartum period [13,15,18,23,24]. However, breastfeeding attitudes were usually established before delivery [50]. Therefore, it may be more important and effective to improve breastfeeding attitudes through interventions that address perinatal depression. Additionally, it is recommended to start the intervention before delivery.

In our study, in addition to general social support, we also considered breastfeeding-specific support (i.e., the level of support for EBF from spouses and other family members). Therefore, we found that other family members’ support for EBF was also a factor of antenatal breastfeeding attitudes. Nevertheless, interestingly, the result of multiple linear regression analyses showed that other family members’ support for EBF was a negative influence on positive breastfeeding attitudes. Pregnant women whose other family members were moderately in favor of EBF had more positive attitudes toward breastfeeding compared to those whose other family members were very supportive of EBF. On the one hand, this may be related to the reverse buffering effect of social support [51]. According to the esteem enhancement theory, when individuals receive social support, they are requested to recognize limitations in their ability to solve problems independently [52,53]. Thus, receiving an overabundance of social support may hurt the recipient’s autonomy and self-reliance, especially if more support is received than given. This in turn damages their independent and capable self-image, which is detrimental to both physical and psychological health [54].Therefore, we hypothesize that the excessive support for EBF from other family members may increase the negative impact of diverse sources of stress (e.g., pregnancy and feeding babies) on maternal mental health, and then negatively affect their breastfeeding attitudes. This needs to be empirically verified in the future studies. On the other hand, we think it may be also related to some traditional cultural factors that are unique to China, such as the relationship between a mother-in-law and daughter-in-law. Practicing traditional confinement periods during the postnatal period is typical in Asian countries [55]. Women are always encouraged to spend a specific period of confinement at their homes, which is considered to be a period of recovery from childbirth [55,56]. During the birth recovery period, most of the maternal caregivers in a family are their mothers-in-law. However, generally, disharmony between a mother-in-law and daughter-in-law is a deep-rooted family conflict in many Asian countries [57]. A poor relationship between mothers-in-law and daughters-in-law is one of the important factors for maternal depression [58]. The strained relationship can easily stimulate the mother’s spirit and emotions, and then they may subconsciously reject the idea that their mother-in-law strongly supports EBF [59]. Therefore, when designing and developing strategies of care around prenatal the promotion of EBF, researchers and health professionals should not only be concerned about the pregnant woman herself, but also about her other family members, especially her mother-in-law. For example, some educational courses that convey tips on how to improve family relationships are necessary.

Additionally, we also found that breastfeeding knowledge was the most important determinant of breastfeeding attitudes. Previous studies have widely reported the significance of breastfeeding knowledge [16,20,60]. The more knowledgeable about breastfeeding, the more positive the attitude towards breastfeeding. Therefore, it is suggested that prenatal education that covers the benefits and skills related to EBF is essential.

Limitations

There are several limitations. First, even though we observed significant results regarding antenatal depressive symptoms and breastfeeding attitudes, the sample size of the study was slightly small. Future research should be focused on larger samples such as a large population-based sample. Second, although we conducted our study using a sample with a low rate of loss to the follow-up, a sampling bias due to the voluntary nature of participation in the study may have affected the findings. For example, we recognized that the underrepresentation of rural pregnant women probably restricted the generalizability of our findings. Third, we controlled as many potential confounding factors as possible based on the literature review. However, there may still be several unobserved variables that may influence the outcome, such as how breastfeeding-friendly the mother’s environment is. Fourth, since we mainly focused on prenatal breastfeeding attitudes, the attitude of these women after childbirth and their actual feeding practices were not described in this study. Additionally, the study was conducted electronically for convenience reasons. Future research needs to consider the refinement of these aspects.

Clinical implications

This study has implications for clinical work in the promotion of EBF, especially the improvement of maternal breastfeeding attitudes. At first, considering that the neutral levels of breastfeeding attitudes and the important determinant role of breastfeeding knowledge, health professionals need to implement education programs for pregnant mothers as early as possible to equip these mothers with positive breastfeeding attitudes. In addition, the early identification and intervention of maternal mental health problems, especially antenatal depressive symptoms, should be implemented to ensure that mothers are motivated to initiate and maintain breastfeeding. Meanwhile, how to adjust and balance the level of support for EBF among the other family members is also something that health professionals must consider when working to improve the breastfeeding attitudes of pregnant women.

## 5. Conclusions

This study showed neutral levels of breastfeeding attitudes among pregnant women in Hunan, China. In addition to the already widely reported breastfeeding knowledge, the determinants of breastfeeding attitudes in our study also included antenatal depressive symptoms and other family members’ support for EBF. Identifying and balancing these modifiable factors that may contribute to poorer or better breastfeeding attitudes may be useful in evaluating the future breastfeeding-promoting interventions. Additionally, in the preliminary analysis, the effect of other family members’ support for EBF and depressive symptoms did not reach statistical significance. However, these two variables were statistically significant in the multiple linear regression. This indicated that the negative effect of other family members’ support for EBF and depressive symptoms might be masked by other factors. In addition, this inconsistency may be also related to the presence of the other potential confounders and mediating or moderating variables between the independent and dependent variables [48,49], which deserves a further exploration.

## Figures and Tables

**Table 1:** Sample characteristics, *n* = 124.

Domain	Characteristics	*n* (%)
Socio-demographic		
	Age, years	
	<35	121 (97.6)
	≥35	3 (2.4)
	Residence	
	Urban	113 (91.1)
	Rural	11 (8.9)
	Race/ethnicity ^a^	
	Han	110 (88.7)
	Minorities	14 (11.3)
	Educational status	
	≤Junior school	2 (1.6)
	High school	10 (8.1)
	Junior college	26 (20.9)
	Bachelor’s degree	75 (60.5)
	≥Master’s degree	11 (8.9)
	Occupation	
	Staff	25 (20.2)
	Self-employed	11 (8.9)
	Medical workers	8 (6.4)
	Farmers	2 (1.6)
	Others	63 (50.8)
	Unemployed	15 (12.1)
	Marital status	
	Not married	6 (4.8)
	Married (live with spouse)	117 (94.4)
	Married (live without spouse)	1 (0.8)
	Household income, CNY	
	<3000 (poor)	3 (2.4)
	3000–5000 (moderate)	24 (19.4)
	>5000 (good)	97 (78.2)
	Pregnancy intention	
	Planned pregnancy	58 (46.8)
	Natural pregnancy	44 (35.5)
	Unplanned pregnancy	22 (17.7)
EBF-related		
	EBF intention	
	Exclusive breastfeeding	49 (39.5)
	Almost exclusive breastfeeding	28 (22.6)
	Partial breastfeeding	44 (35.5)
	Token breastfeeding	2 (1.6)
	Exclusive artificial feeding	1 (0.8)
	Spousal support for EBF	
	Very supportive	61 (49.2)
	Support	53 (42.7)
	Moderate	10 (8.1)
	Not supported	0 (0.0)
	Very unsupported	0 (0.0)
	Other family members’ support for EBF	
	Very supportive	64 (51.6)
	Support	56 (45.2)
	Moderate	4 (3.2)
	Not supported	0 (0.0)
	Very unsupported	0 (0.0)

Note: ^a^ There are 56 ethnic groups in China. In addition to the largest number of people, the Han Chinese, there are 55 officially recognized minorities. Abbreviations: SD, standard deviation. EBF, Exclusive Breastfeeding.

**Table 2:** Distribution of the mean scores the participants obtained from scales, *n* = 124.

	Scales	Mean	SD	Minimum	Maximum
1	IIFAS	56.39	5.69	45	70
2	EPDS	6.45	3.99	0	22
3	PSRS	19.36	13.77	0	61
4	CAQ	35.90	10.30	16	64
5	BKQ	10.56	4.40	0	17
6	PSSS	69.87	11.14	16	84

Note: Abbreviations: SD, standard deviation. IIFAS, the Iowa Infant Feeding Attitude Scale. EPDS, Edinburgh Postnatal Depression Scale. PSRS, Pregnancy Stress Rating Scale. CAQ, childbirth attitudes Questionnaires. BKQ, Breastfeeding Knowledge Questionnaire. PSSS, Perceived Social Support Scale.

**Table 3:** Univariate analysis of factors associated with breastfeeding attitudes, *n* = 124.

Domain	Characteristics	Mean ± SD	t/F	*p*
Socio-demographic				
	Age, years		0.529	0.598
	<35	56.43 ± 5.74		
	≥35	54.67 ± 2.31		
	Residence		−1.721	0.088
	Urban	56.12 ± 5.64		
	Rural	59.18 ± 5.64		
	Race/ethnicity ^a^		1.323	0.188
	Han	56.63 ± 5.71		
	Minorities	54.50 ± 5.30		
	Educational status		7.693	0.103
	≤Junior school	55.00 ± 0.00		
	High school	55.10 ± 6.32		
	Junior college	54.35 ± 5.00		
	Bachelor’s degree	56.96 ± 5.40		
	≥Master’s degree	58.73 ± 7.89		
	Occupation		4.435	0.489
	Staff	57.16 ± 6.26		
	Self-employed	56.55 ± 5.72		
	Medical workers	59.88 ± 5.44		
	Farmers	56.50 ± 0.71		
	Others	55.81 ± 5.60		
	Unemployed	55.53 ± 5.44		
	Marital status		3.688	0.158
	Not married	53.83 ± 4.45		
	Married (live with spouse)	56.43 ± 5.67		
	Married (live without spouse)	67.00 ± 0.00		
	Household income, CNY		5.119	0.077
	<3000 (poor)	64.67 ± 5.13		
	3000–5000 (moderate)	56.67 ± 5.84		
	>5000 (good)	56.06 ± 5.52		
	Pregnancy intention		2.223	0.329
	Planned pregnancy	56.55 ± 5.59		
	Natural pregnancy	56.84 ± 5.86		
	Unplanned pregnancy	55.05 ± 5.65		
EBF-related				
	EBF intention		10.417	0.034 *
	Exclusive breastfeeding	57.90 ± 5.57		
	Almost exclusive breastfeeding	55.79 ± 5.38		
	Partial breastfeeding	55.14 ± 5.68		
	Token breastfeeding	51.50 ± 4.95		
	Exclusive artificial feeding	64.00 ± 0.00		
	Spousal support for EBF		5.327	0.070
	Very supportive	57.61 ± 5.79		
	Support	55.30 ± 5.08		
	Moderate	54.70 ± 6.99		
	Other family members’ support for EBF		4.427	0.109
	Very supportive	57.39 ± 5.89		
	Support	55.13 ± 4.95		
	Moderate	58.00 ± 9.70		

Note: ^a^ There are 56 ethnic groups in China. In addition to the largest number of people, the Han Chinese, there are 55 officially recognized minorities. Breastfeeding attitudes were measured using the Iowa Infant Feeding Attitude Scale. Abbreviations: SD, standard deviation. EBF, exclusive breastfeeding. * *p* < 0.05.

**Table 4:** Correlation analysis of the variables, *n* = 124.

	Variable	1	2	3	4	5
1	Breastfeeding attitudes	-				
2	Depressive symptoms	−0.134	-			
3	Pregnancy stress	−0.126	0.459 ***	-		
4	Fear of childbirth	−0.082	0.354 ***	0.503 ***	-	
5	Breastfeeding knowledge	0.505 ***	−0.050	−0.073	−0.087	-
6	Social support	0.145	−0.331 ***	−0.340 ***	−0.106	−0.233 **

Note: ** *p* < 0.01, *** *p* < 0.001.

**Table 5:** Multiple linear regression analyses of determinants of breastfeeding attitudes, *n* = 124.

Dependent Variable		B	SE	β	T	*p*
Breastfeeding attitudes	Constant	55.212	5.201		10.616	<0.001 ***
	Residence					
	Urban	Reference				
	Rural	2.598	1.679	0.130	1.547	0.125
	Educational status					
	≤Junior school	Reference				
	High school	4.052	3.850	0.195	1.052	0.295
	Junior college	2.599	3.549	0.187	0.732	0.466
	Bachelor’s degree	4.221	3.502	0.364	1.205	0.231
	≥Master’s degree	6.934	3.790	0.348	1.829	0.070
	Household income, CNY					
	<3000 (poor)	Reference				
	3000–5000 (moderate)	−3.703	3.086	−0.258	−1.200	0.233
	>5000 (good)	−5.748	2.985	−0.419	−1.926	0.057
	EBF intention					
	Exclusive breastfeeding	Reference				
	Almost exclusive breastfeeding	−2.256	1.219	−0.167	−1.851	0.067
	Partial breastfeeding	−1.633	1.130	−0.138	−1.446	0.151
	Token breastfeeding	−4.878	4.094	−0.109	−1.192	0.236
	Exclusive artificial feeding	6.892	4.809	0.109	1.433	0.155
	Spousal support for EBF					
	Very supportive	Reference				
	Support	−1.486	1.715	−0.130	−0.866	0.388
	Moderate	−3.521	2.311	−0.169	−1.524	0.131
	Other family members’ support for EBF					
	Very supportive	Reference				
	Support	0.629	1.688	0.055	0.373	0.710
	Moderate	8.922	3.403	0.278	2.622	0.010 *
	Depressive symptoms	−0.272	0.114	−0.191	−2.381	0.019 *
	Breastfeeding knowledge	0.562	0.113	0.434	4.966	<0.001 ***
	Social support	−0.009	0.044	−0.017	−0.195	0.846

Note: Abbreviations: EBF, exclusive breastfeeding. R = 0.660, R^2^ = 0.436, Adjusted R^2^ = 0.339, F = 4.507, *p* < 0.001. * *p* < 0.05, *** *p* < 0.001.

## Data Availability

Not applicable.
